# Pavlovian fear memory induced by activation in the anterior cingulate cortex

**DOI:** 10.1186/1744-8069-1-6

**Published:** 2005-02-09

**Authors:** Jianrong Tang, Shanelle Ko, Hoi-Ki Ding, Chang-Shen Qiu, Amelita A Calejesan, Min Zhuo

**Affiliations:** 1 Department of Physiology, Faculty of Medicine, University of Toronto Centre for the Study of Pain, University of Toronto, Medical Sciences Building, Rm 3342, 1 King's College Circle, Toronto, ON M5S 1A8, Canada

## Abstract

Identifying higher brain central region(s) that are responsible for the unpleasantness of pain is the focus of many recent studies. Here we show that direct stimulation of the anterior cingulate cortex (ACC) in mice produced fear-like freezing responses and induced long-term fear memory, including contextual and auditory fear memory. Auditory fear memory required the activation of N-methyl-D-aspartate (NMDA) receptors in the amygdala. To test the hypothesis that neuronal activity in the ACC contributes to unpleasantness, we injected a GABA_A _receptor agonist, muscimol bilaterally into the ACC. Both contextual and auditory memories induced by foot shock were blocked. Furthermore, activation of metabotropic glutamate receptors in the ACC enhanced behavioral escape responses in a noxious hot-plate as well as spinal nociceptive tail-flick reflex. Our results provide strong evidence that the excitatory activity in the ACC contribute to pain-related fear memory as well as descending facilitatory modulation of spinal nociception.

## Background

Pain in humans is an unpleasant sensory and emotional experience associated with actual or potential tissue damage, or described in terms of such damage [1]. The pain experience contains at least two major components: the first is the encoding and perception of sensory noxious stimulus (e.g., pain intensity); and the second is the encoding of the unpleasantness of the noxious stimuli [2,3]. Exploration of the centers for pain-related unpleasantness has recently been carried out in human studies using modern imaging techniques [4-8]. Among many central regions investigated, the anterior cingulate cortex (ACC) is believed to be a key structure that contributes to pain affect or unpleasantness. Early human observations showed that surgical ablation of the ACC significantly reduced pain unpleasantness without influencing the ability to detect the intensity or location of the pain [9,10]. Rainelle et al [5] reported that specific manipulation of pain unpleasantness produced significant changes in the imaged activity of the ACC, while the manipulation of pain intensity produced changes mainly in the primary somatosensory cortex (S1) [2,5]. More recently, electrophysiological recordings from the ACC in humans found that some ACC neurons respond to noxious stimuli [6]. More interestingly, a recent study reported that the ACC was also activated during social exclusion [11]. In addition to pain, the ACC has been proposed as the neurobiological substrate for executive control of cognitive and motor processes [12]. Human imaging studies demonstrate that the ACC region is activated by different factors including motivational drive, reward, gain or loss, conflict-monitoring or error prediction, and attention or anticipation [13-23]. The neuronal mechanisms for these different functions within the ACC remain mostly unknown due to the limitation of human studies.

Studies from our group and other investigators, using animal models, provide evidence for the importance of the ACC in behavioural responses related to noxious stimuli [24-32] and the "top-down" descending modulatory effects [33]. Lesion in the medial frontal cortex, including the ACC, significantly reduced the behavioral response to noxious stimuli and aversive memory behaviors [24-26]. Also, electrophysiological recordings demonstrate that neurons within the ACC respond to noxious stimuli [6,28]. Tissue injury or digit amputation activates immediate early gene expression and triggers long-term potentiation of evoked sensory responses in the ACC [27-29]. In mice genetically modified to over express NMDA NR2B receptors in forebrain areas, including the ACC, behavioral responses to tissue inflammation were significantly enhanced [29]. Behavioural allodynia related to inflammation was reduced by injection of antagonists of NMDA receptors or inhibitors of cAMP-dependent protein kinases [30,32]. These findings indicate that ACC neurons are clearly involved in the processing of noxious stimuli, and demonstrate activity-dependent long-term plasticity in the ACC after tissue injury. In addition, ACC can also serve as a "top-down" descending modulatory system that regulates spinal nociceptive reflexes. Electrical stimulation or chemical injection of glutamate receptor agonists facilitated a spinal nociceptive tail-flick reflex through a descending facilitatory system relayed to the brainstem rostral ventromedial medulla (RVM) [33-37].

It is difficult to distinguish the role of the ACC in pain-related unpleasantness from its descending pain modulatory effects on sensory transmission in the spinal cord by using behavioral withdrawal responses to noxious stimuli. A recent human imaging study reported that the ACC is activated during placebo analgesia [7]. These results suggest that the ACC may also play roles in placebo analgesia. While the physiological nature of the imaged 'hot' spots (i.e., excitation of excitatory versus inhibitory neurons) remains to be determined, it has been proposed that the ACC may activate endogenous analgesia systems due to its projections to the periaqueductal gray (PAG) in the midbrain [7,38].

Here we propose that the ACC serves as a region for pain unpleasantness in the brain, and excitation of neurons in the ACC can trigger pain unpleasantness but not analgesia. Because animals will never report human-like 'unpleasantness', we used a classic Pavlovian fear memory to measure the effects of stimulation in the ACC. Our operational definition of 'unpleasantness' in mice is based on the formation of fear memory. If ACC stimulation triggers 'unpleasantness' in mice, we expect to observe behavioural freezing responses in mice receiving paired but not unpaired fear-conditioning training. Another obvious advantage of using the Pavlovian fear memory model is to avoid using behavioural withdrawal responses (e.g., hind paws or tail) that are constantly under descending inhibitory and facilitatory modulatory influences. Experiments were performed in both mice and rats to test the hypothesis.

## Results

**Figure 1:**
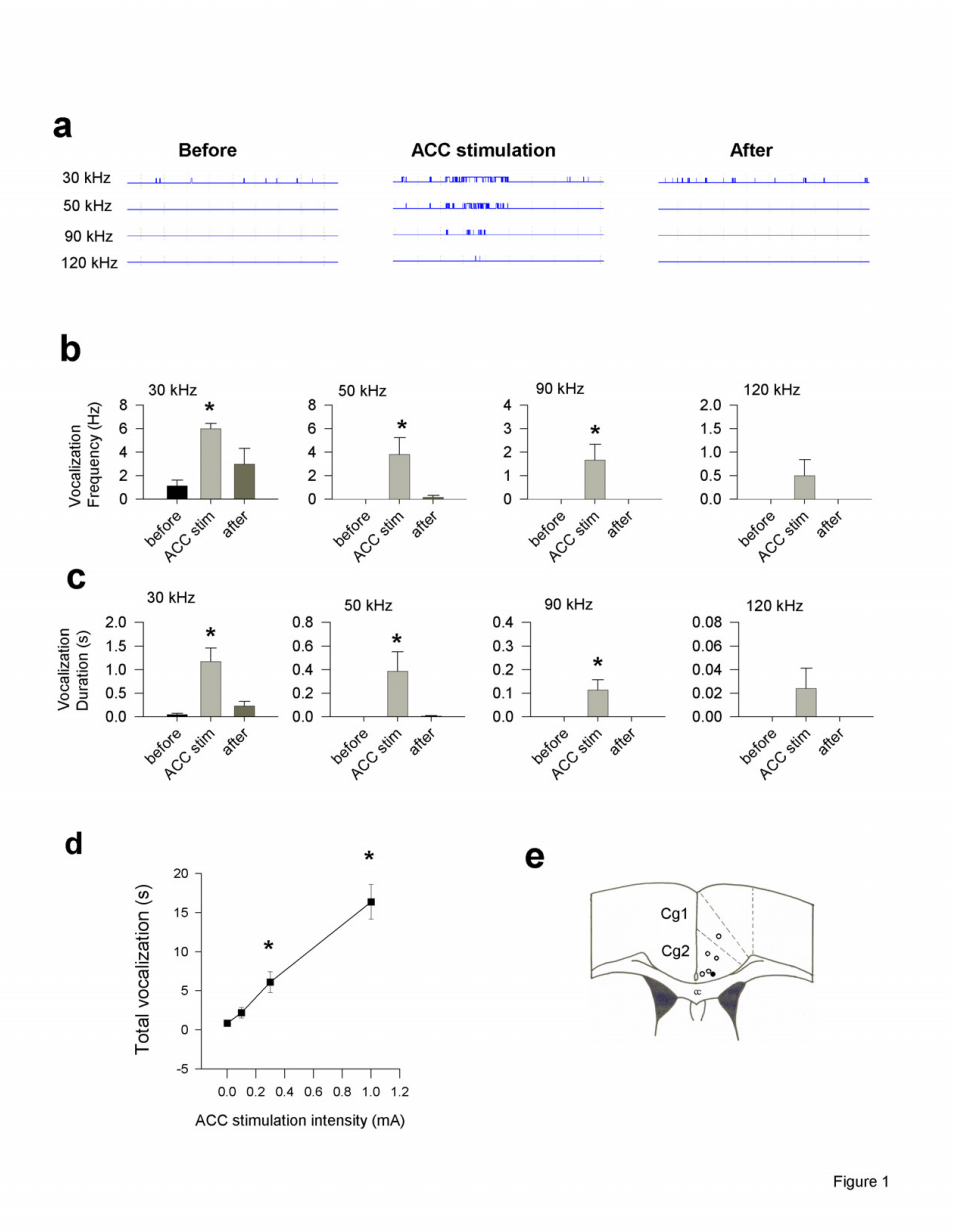
**ACC stimulation induces ultrasonic vocalization in freely moving mice. (a) **An example of ultrasonic responses from a single mouse at four different frequencies before, during and after ACC stimulation (at 0.3 mA). 1 min duration; see filled circle for the stimulation site within the ACC in (e); **(b) **ACC stimulation (0.3 mA; n = 6) increased the frequency of individual ultrasonic responses; * *P *<*0.05*, comparing the frequency during ACC stimulation with baseline response before the stimulation; **(c) **ACC stimulation (0.3 mA; n = 6) also increased the duration of single ultrasonic response; * *P < 0.05*, comparing the duration during ACC stimulation with baseline duration before the stimulation; **(d) **Summarized data of ACC stimulation (n = 6) produced ultrasonic responses at different intensities. Total vocalization responses (sec) within 2 min ACC stimulation were plotted against the intensity of stimulation; **(e) **Stimulating sites in ACC on the schematic representation of coronal section 0.62 mm anterior to the Bregma. Filled circle, for data shown in (a); open circles, other sites for data shown in (b-d).

We employed direct focal electrical stimulation of the ACC in freely moving adult mice and used fear memory to test if the ACC encodes for pain-related unpleasantness. Animals were implanted with stimulating electrodes in the ACC, and behavioural measurements were performed at least 2 weeks after the surgery. Mice were placed in a specially designed sound insulated chamber. After at least 30 min of baseline observation, a brief electrical current was delivered to the ACC through the implanted electrodes. We monitored ultrasonic activity, another index of emotional responses [39], in some of the mice throughout the experiments. Ultrasonic responses were increased during ACC stimulation at 0.3 mA (n = 6 mice; *P *<*0.05 *as compared with stimulation at 0 mA; see Fig. 1). Significant changes were detected for both the frequency and duration of ultravocalization events (Fig. 1b and 1c). The ultrasonic activity induced by electrical stimulation was intensity dependent. Stimulation at 0.01 mA produced almost no significant changes, while stimulation at 1.0 mA induced greater responses (n = 6 mice, *P *<*0.01 *as compared with stimulation at 0.1 or 0.3 mA; Fig. 1d). Ultrasonic activity was found mostly between 30–50 kHz. This frequency range of ultrasonic activity was also increased by a noxious foot shock and by a painful chemical injection of capsaicin. No obvious changes in motor activity or seizure-like activity were found in mice after receiving the focal ACC stimulation.

**Figure 2:**
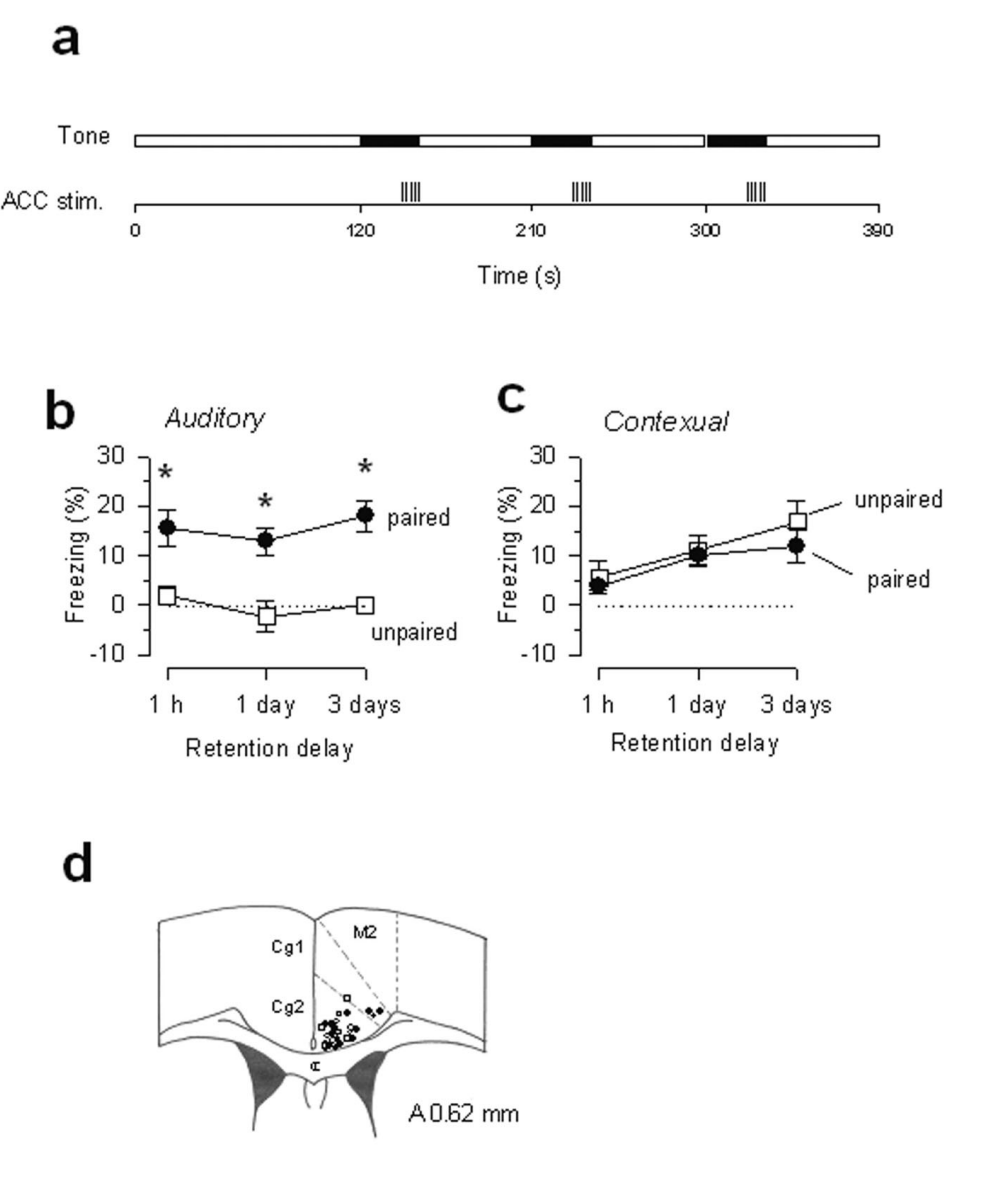
**ACC stimulation induces long-term fear memory. (a) **Three pairings of 30 s tone and 10 s electrical train stimulation were delivered in the paired group on the conditioning day; **(b**,**c) **Percentage freezing to the tone (**b**) and the conditioning context (**c**) measured at 1 hr, 1 day and 3 days after paired training. After paired training, long-lasting fear memory was detected in most of the mice (n = 16 mice, filled circles), while some other mice showed no freezing across the test periods (n = 5, data not shown). After unpaired training of tone and ACC stimulation, mice showed no freezing to the tone (n = 6, open squares) but clear memory to the training environment; * *P < 0.05 *compared with the unpaired group;**(d) **Stimulating sites in ACC on the schematic representation of coronal section 0.62 mm anterior to the Bregma. Filled circles, effect sites of ACC paired group; open circles, no effect sites of ACC paired group; open squares, ACC unpaired group.

Next, we wanted to test if the fear-like freezing responses induced by ACC stimulation were due to unpleasantness (similar to that caused by a noxious foot shock). If so, we would expect to see long-term fear memory induced by ACC stimulation. We paired ACC electrical stimulation with a tone presented in a conditioning chamber (see Fig. 2a). After pairing ACC stimulation with the tone, long-term fear memory was detected in most of the mice (n = 16 of 21 mice; 76.2%), both to the tone presentation in a novel context (auditory memory) (Fig. 2b) and to the conditioning environment (contextual memory) (Fig. 2c). In the other animals, there is no specific freezing either to the conditioning context or to the tone (Fig. 2d; n = 5 mice). We did not find any clear anatomic separation between effective versus ineffective stimulation sites in the ACC for producing fear memory (Fig. 2d). There are two possible explanations for this result. One is that some of sites may have a higher stimulation threshold for inducing fear memory. Alternatively, ACC neurons are functionally heterogeneous and some sites within the ACC do not contribute to the unpleasantness of pain.

Pavlovian fear memory occurs when a subject learns to associate a certain conditioned stimulus (CS) or cue with a noxious unconditioned stimulus (US) [40-43]. To determine whether ACC stimulation-induced fear memory results from association between the tone and ACC stimulation, we performed experiments where the tone and ACC stimulation were applied but unpaired in another group of animals (Fig. 2b–c). We predicted that while similar contextual memory may form (since animals received the same amount of ACC stimulation in the same environment), auditory memory, which requires the precise pairing of US-CS, would be blocked. Indeed, mice receiving unpaired training demonstrated clear freezing response in the conditioning context (n = 6 mice; *P < 0.05 *versus baseline responses), whereas no freezing response was observed during tone presentation in the novel context (*P > 0.05*, comparing behavioral responses before and during the tone; Fig. 2b-–c).

**Figure 3:**
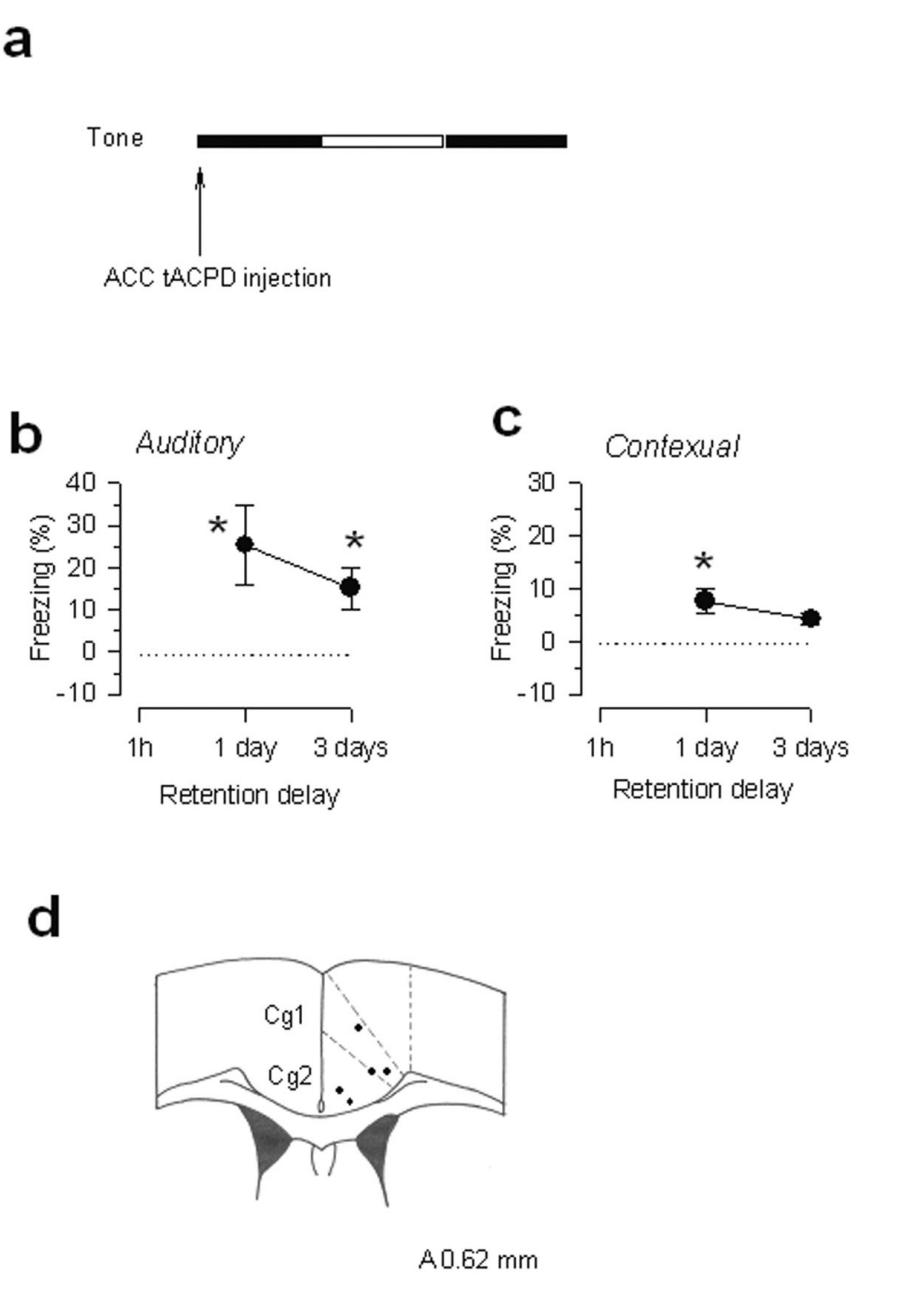
**Activation of metabotropic glutamate receptors in the ACC caused long-term fear memory. (a) **ACC tACPD microinjection (0.25 μg in 0.5 μl; indicated by an arrow) was paired with a tone (indicated by filled bar) on the conditioning day; **(b, c) **Percentage freezing to the tone (**b**) and the conditioning context (**c**) measured at 1 day and 3 days after paired training (n = 5 mice). *P < 0.05 compared with the control. **(d) **The sites in the ACC for tACPD microinjection on the schematic representation of coronal section 0.62 mm anterior to the Bregma.

Glutamate receptors including metabotropic glutamate receptors (mGluRs) are found in the ACC [33,38]. To further determine if local activation of glutamate receptors in the ACC may also trigger similar responses as focal electrical stimulation, we performed microinjection of a mGluR agonist tACPD (0.25 μg in 0.5 μl) [33]. Microinjection of tACPD into the ACC produced freezing responses within 10 min, indicating that activation of ACC neurons induced freezing responses (n = 5 mice). Furthermore, we also paired the tACPD microinjection with a tone (see Fig. 3a) to test if tACPD application with tone may also cause fear memory. We measured fear responses at one and three days the conditioning. We found that tACPD microinjection pairing produced significant behavioral freezing responses at one and three days later (n = 5 mice; Fig. 3b and 3c). Mice receiving the tACPD microinjection in the ACC did not cause any visible abnormal motor hyperactivity or seizure-like behaviors, although we cannot completely rule out other sub-threshold changes caused by the microinjection.

**Figure 4:**
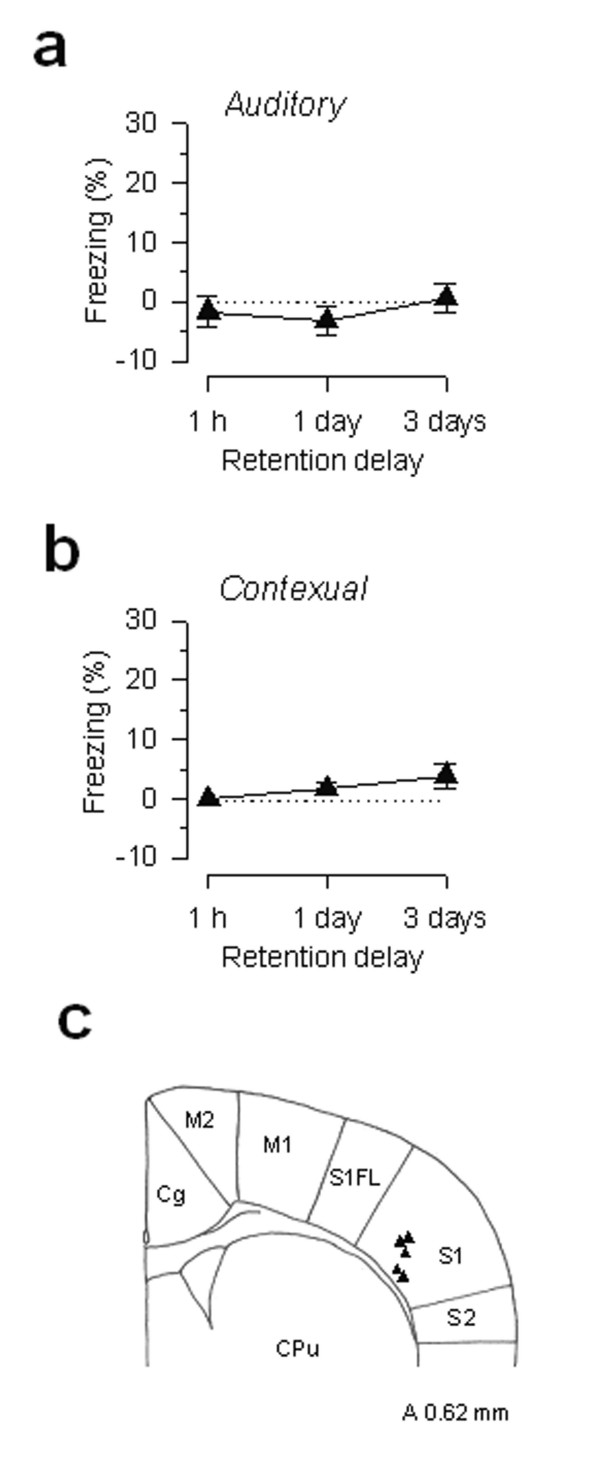
**Primary somatosensory cortex (S1) stimulation produces no long-term fear memory. (a, b) **Three pairings of 30 s tone and 10 s electrical train stimulation were delivered to somatosensory cortex (S1) caused neither long-term auditory (a) nor contextual memory (b) (n = 5). **(c) **Sites for stimulation in the S1.

Next, we asked whether stimulation-induced unpleasantness is regional selective. If ACC activation is specifically involved in fear conditioning, electrical stimulation outside of the ACC would not produce similar results. To test this possibility, stimulating electrodes were implanted into the primary somatosensory cortex (S1). It has been well documented that somatosensory cortex contributes to the central processing of sensory inputs, including pain intensity (e.g., noxious stimulus parameters) [44]. As shown in Fig. 4, this group of mice did not form fear memory, even though they underwent exactly the same conditioning procedures as the paired groups (*P > 0.05 *versus baseline; n = 5 mice).

**Figure 5:**
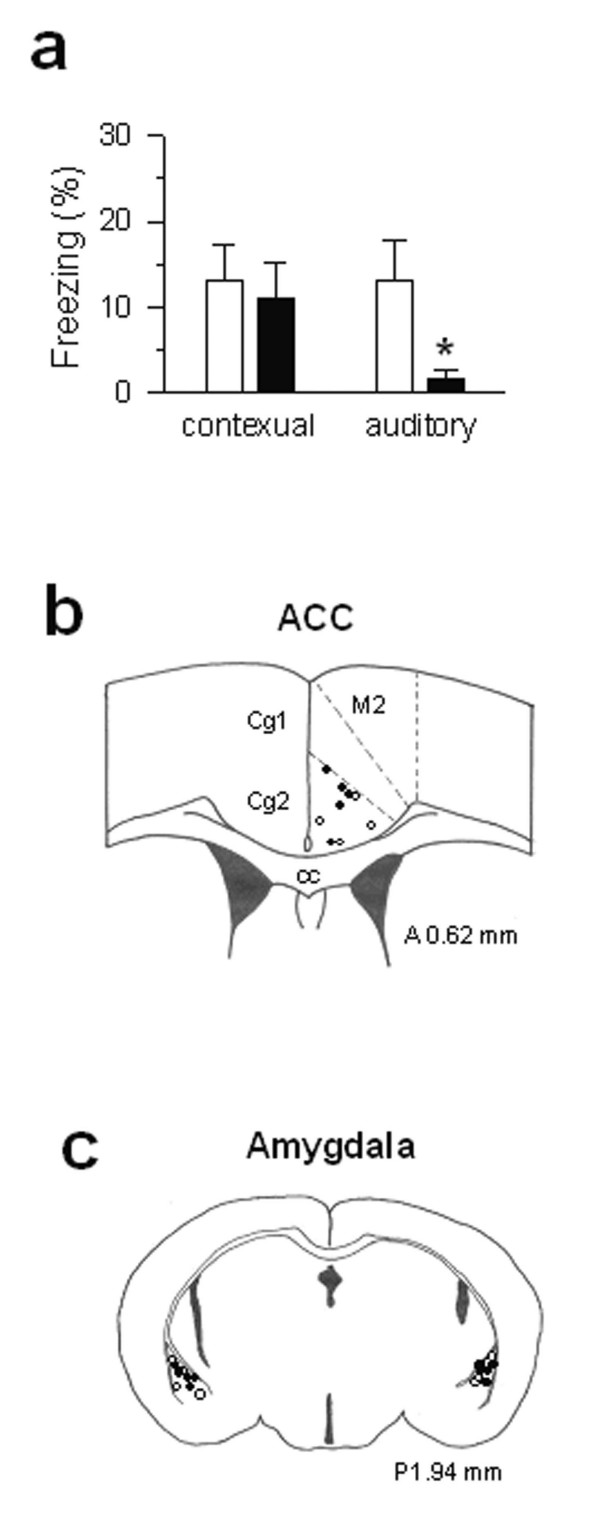
**NMDA receptors in the amygdala is required for auditory fear memory induced by ACC stimulation. (a) **Bilateral microinfusions of AP-5 (2 μg/μl, 0.5 μl/side) in the BLAC 15 min before conditioning impaired auditory fear memory but no effect on contexual fear memory when tested 1 day later. Filled bars, AP-5 group (n = 5); open bars, saline group (n = 4). * P < 0.05 compared with saline-injected group. **(b) **Stimulation sites in ACC. Filled circles, AP-5 group; open circles, saline group. **(c) **Microinjection sites in the BLAC on the schematic representation of coronal section 1.94 mm posterior to the Bregma. Filled circles, AP-5 group; open circles, saline group.

Amygdala and its related structures are well known for the induction of fear memory [40-43]. While the prefrontal cortex has been recently implicated for its involvement in the extinction of fear memory [45-47], it has not been shown that ACC inputs to the amygdala may contribute to the formation of fear memory. To test if fear-like long-term memory induced by ACC conditioning requires the involvement of amygdala, we selectively blocked the NMDA receptors during training (ACC stimulation paired with the environment or a tone). The NMDA receptors are known to be important for the induction of fear memory [48,49]. We implanted additional guide cannulas into the basolateral amygdaloid complex (BLAC) for the microinjection of drugs (Fig. 5c). Before conditioning, the NMDA receptor antagonist AP-5, was injected bilaterally into the BLAC. At 15 min later, ACC stimulation was paired with the tone presentation. For the control group, the same volume of saline was injected. As showed in Fig. 5a, AP-5 significantly reduced ACC stimulation-induced fear memory to the tone 1 day after conditioning (*P < 0.05 *versus saline injected group; n = 5 mice), indicating that the NMDA receptor function in the amygdala is required for the formation of auditory fear memory. Importantly, the contextual memory was not affected (n = 5 mice; Fig. 5a), supporting the notion that other structures such as the hippocampus may contribute to contextual fear memory.

**Figure 6:**
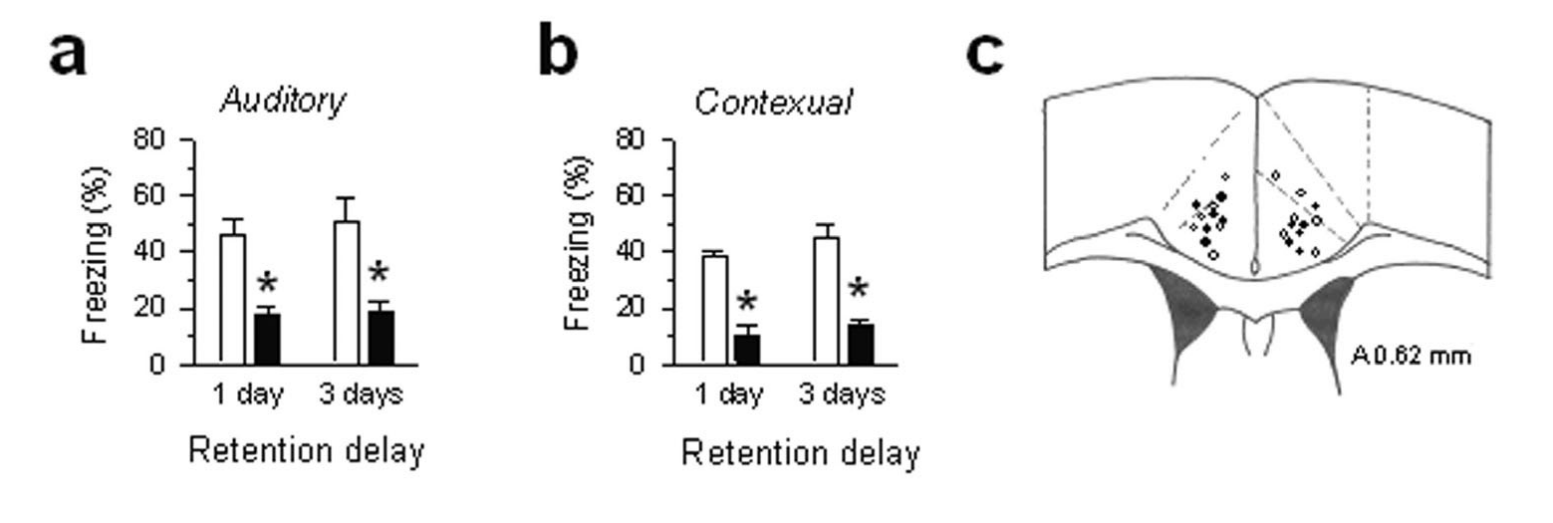
**ACC inactivation by a GABA_A _receptor agonist impairs fear memory by foot shock. (a**,**b) **Mice receiving muscimol microinjection (1 μg/μl, 0.5 μl/each side, n = 6 mice, black bars) into bilateral ACC 15 min before conditioning showed reduced auditory (a) and contextual (b) fear memory induced by classic foot shock conditioning). * *P < 0.05 *compared with the saline treated group (n = 6, open bars). **(c) **Microinjection sites in the ACC. Filled circles: muscimol group; open circles, saline group.

These results suggest that ACC stimulation produces unpleasantness in mice and form fear auditory memory through its interaction with the amygdala. Both animal and human studies indicate that neurons in the ACC respond to peripheral painful stimuli [6,28,50], and injury triggers the activation of immediate early genes in the ACC [27,29]. Unlike the somatosensory cortex, neurons in the ACC have wide diffuse receptive fields, and often contain the whole body of an animal, supporting its role in coding unpleasantness of pain [6,51,52]. Supporting this hypothesis, lesions of the ACC or inhibition of excitatory synaptic transmission in the ACC produces antinociceptive effects or analgesia [24,25] and block formalin-induced conditioned place avoidance [26]. These results argue that activation of the ACC or increased the excitatory activity within the ACC is unpleasant or painful, acting directly or indirectly through other related brain regions. However, a recent imaging study reported that the activity in the ACC was increased during placebo analgesia [7]. We think that the activation of endogenous analgesia system is unlikely to account for placebo analgesia. Previous studies showed that stimulation of ACC did not trigger the endogenous analgesia system [33]. Rather, we propose that enhanced inhibitory activity may contribute to the placebo effects. To test if increasing inhibitory transmission in the ACC may relieve unpleasantness due to foot shock, we microinjected a GABA_A _receptor agonist, muscimol into the ACC prior to the fear conditioning. Muscimol significantly reduced the fear memory score 1 day and 3 days after classical fear conditioning (*P < 0.05 *versus saline injected group, n = 6 mice for each group) (Fig. 6a–c).

**Figure 7:**
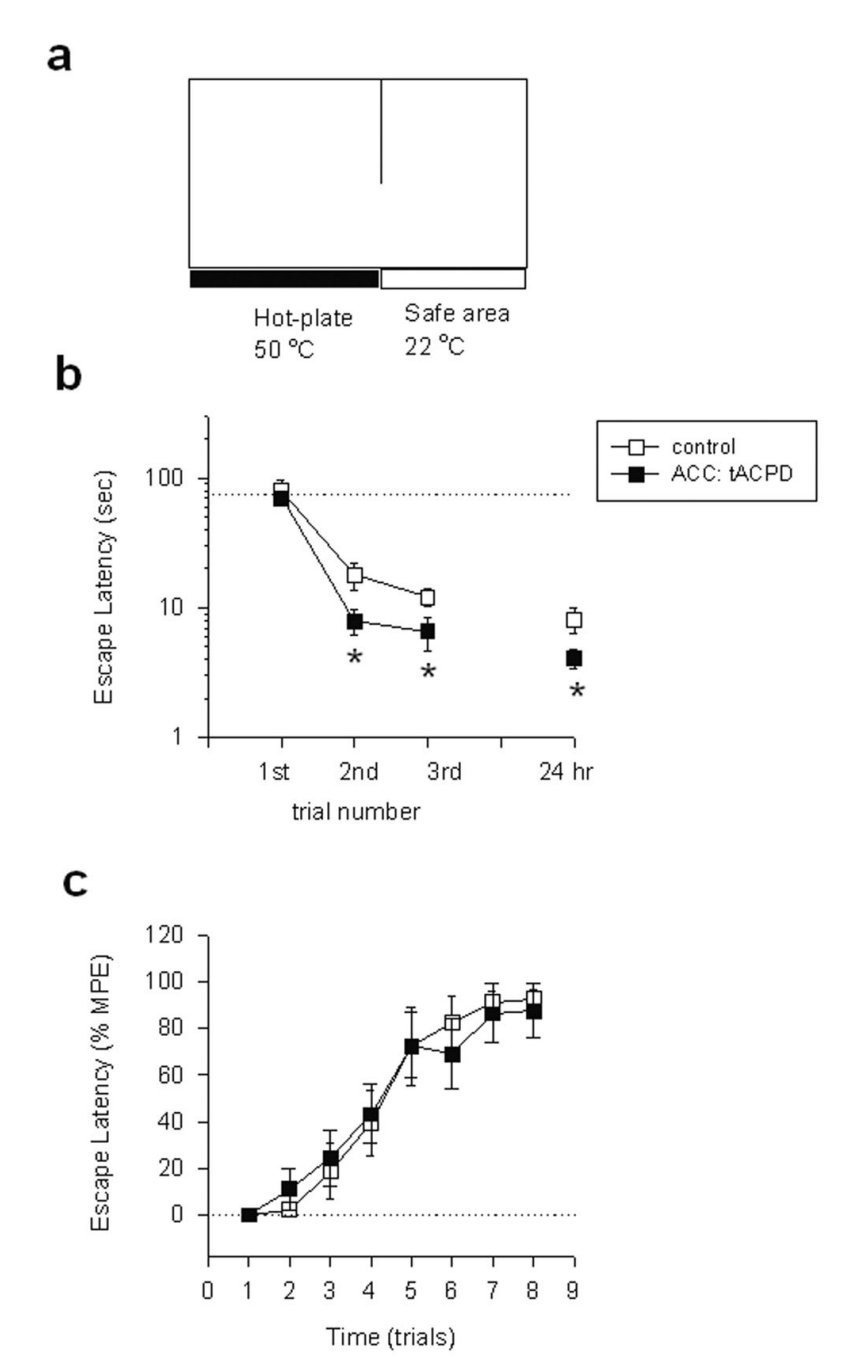
**Activation of mGluRs in the ACC facilitated escaping behavioral responses in a hot-plate. (a) **A diagram explaining a new behavioural escape test using the modified hot-plate instrument. **(b) **Mice receiving tACPD microinjection into the unilateral ACC 10 min before training showed faster escape response. * *P < 0.05 *compared with the control group (n = 6 mice, open squares). **(c) **Extinction responses in control and tACPD-treated groups were similar. MPE were calculated as: (response latency – baseline latency)/(180 – baseline latency). 100% MPE indicates that mouse stayed in the same plate for 3 min without moving into the safe area.

One possible explanation is that activation of ACC neurons is aversive in the absence of peripheral noxious stimuli, and is antinociceptive in case of peripheral noxious stimuli. To test this possibility, we decided to test if activation of the ACC alters animals' avoidance responses using the modified noxious hot-plate escape test. In this test, mice learn to escape the noxious area by moving into an unheated area. In the first trail, mice found the escape route to the unheated area within 79.6 ± 17.7 sec after being placed on the 50°C hot-plate, (n = 7 mice, Fig. 7a). After repetitive training (n = 3 in total, with a 10 min interval), mice learned to move into the 'safe' area within a significantly shorter period of time (12.1 ± 1.8 sec; *P < 0.01 *compared with the first measurement). Furthermore, at 24 hours after training, mice quickly moved into the safe area within a similar time (mean 8.1 ± 1.8 sec, n = 7 mice; Fig. 7b). Next, we wanted to test if activation of the ACC may affect behavioral escape responses. tACPD (0.25 μg in 0.5 μl) was microinjected into the ACC before the training. While the escape time at the first trial was not significantly affected by tACPD microinjection (n = 7 mice, mean 70.1 ± 8.0 sec), mice moved into the safe area within a significantly shorter period of time during the second and third trials (*P < 0.01 *as compared with control mice). Furthermore, at 24 hours after the training, mice moved into the safe area significantly faster than control mice (*P < 0.05*, Fig. 7b). To determine if the effects of tACPD may affect subsequent extinction, we repetitively exposed both groups of mice to the same plate at room temperature (22°C) at 30 min intervals. Mice spent significantly longer time in the plate after learning that the plate was not hot or noxious (n = 7 mice; Fig. 7c). As shown in Fig. 7c, both groups of animals showed similar 'learning' responses. These results indicate that local activation of mGluRs in the ACC selectively enhanced the learning ability to avoid the noxious plate.

**Figure 8:**
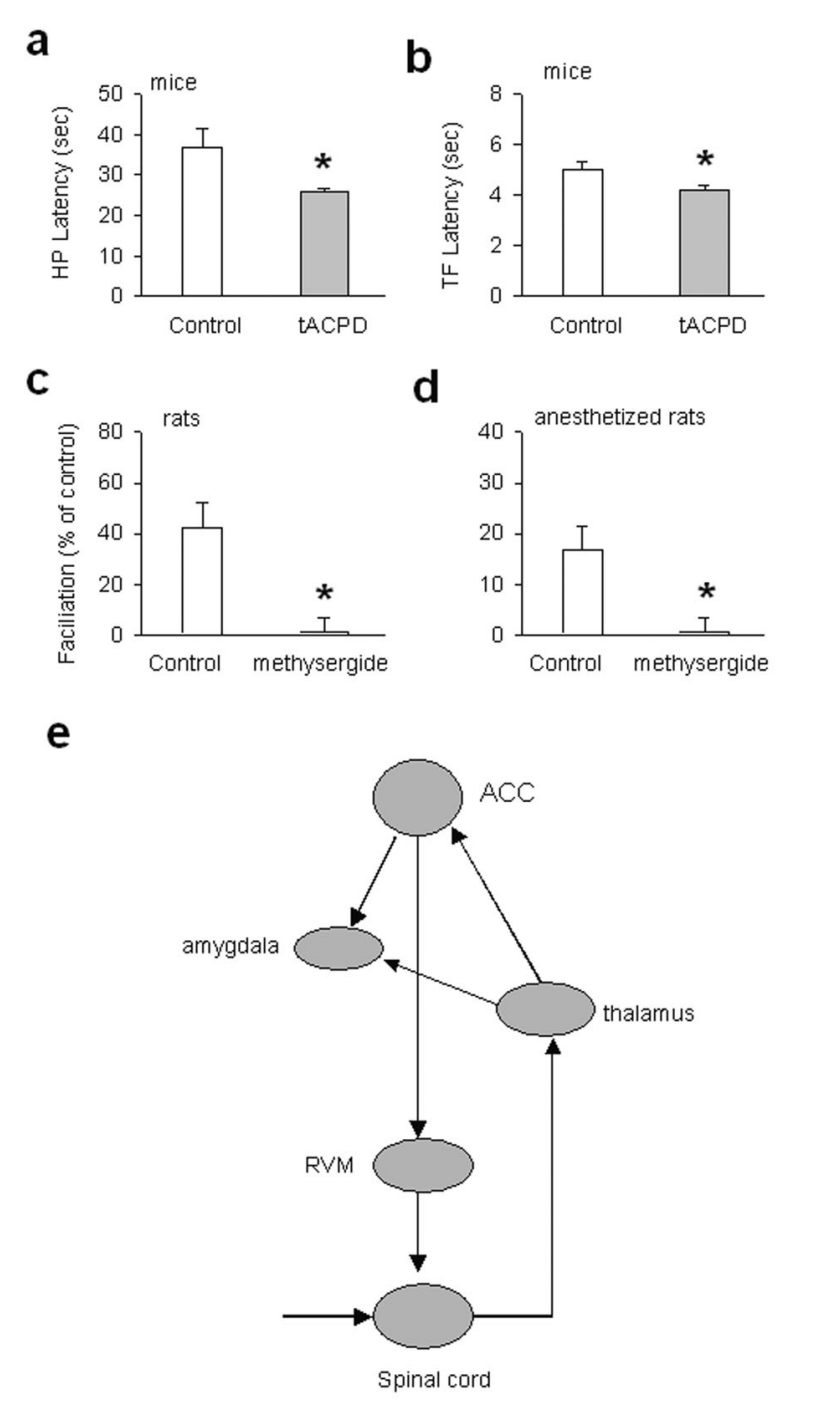
**Spinal serotonin receptors contribute to descending facilitatory modulation from the ACC. (a, b) **Microinjection of tACPD (0.25 μg in 0.5 μl) in the ACC facilitated the hot-plate responses (a, i.e., reduced response latency) and spinal nociceptive tail-flick reflex (b) in freely moving mice; **(c, d) **In freely moving (c) or anesthetized rats (d), tACPD microinjection in the ACC also facilitated the tail-flick reflex. Intrathecal injection of a serotonergic receptor antagonist methysergide (32.0 nmoles/10 μl) at a dose that blocked descending facilitation also blocked the facilitation of the tail-flick reflex.**(e) **A model explaining the neuronal pathways, which contribute to ACC activation, produced fear memory and descending facilitatory modulation of spinal nociception.

To examine the effects of microinjection of tACPD in the ACC on behavioral nociceptive responses, we examined the effects of chemical activation of mGluRs in the ACC on the spinal nociceptive tail-flick reflex and the hot-plate test in awake mice. Microinjection of tACPD into the ACC produced facilitation of spinal tail-flick reflex (n = 7 mice; Fig. 8b). Furthermore, the response latency in the hot-plate test was also significantly reduced (n = 5 mice, Fig. 8a). These results consistently suggest that activation of mGluRs in the ACC is able to facilitate or enhance behavioral responses to noxious stimuli. Previous studies in anesthetized rats demonstrate that descending facilitation induced by electrical/chemical activation in the ACC depend on central relays in the brainstem RVM [33]. Descending serotonergic systems originated from the brainstem raphe nucleus as well as adjacent areas are thought to be important for descending facilitation of spinal nociceptive transmission and reflexes [34-37]. To examine if spinal serotonergic receptors are required for tACPD-induced facilitation, we directly injected a serotonergic receptor antagonist into the spinal cord through intrathecal catheters in freely moving adult rats. Similar to adult mice, microinjection of tACPD into the ACC produced a significant reduction in the tail-flick responses latencies in rats (n = 3 rats, mean 5.8 ± 0.8 sec vs 3.2 ± 0.1 sec, *P < 0.01*, paired t-test). Intrathecal administration of methysergide at a dose of 32.0 nmoles (10 μl, i.th.) that blocked descending facilitatory effects from the RVM [34] completely abolished the facilitation induced by tACPD in the ACC (n = 3 rats; Fig. 8c). To avoid possible side effects of stress, we also performed experiments in anesthetized rats. As reported previously [33], microinjection of tACPD produced significant facilitation of the tail-flick response latency in rats (n = 4 rats, mean 6.3 ± 0.3 sec vs 5.3 ± 0.5 sec, *P < 0.05*; Fig. 8d). Methysergide (32.0 nmoles/10 μl) injected intrathecally blocked the facilitatory effects produced by tACPD in the ACC (n = 4 mice, Fig. 8d). These results consistently indicate that activation of the ACC causes facilitation or anti-analgesic effects in case of noxious stimulation.

**Figure 9:**
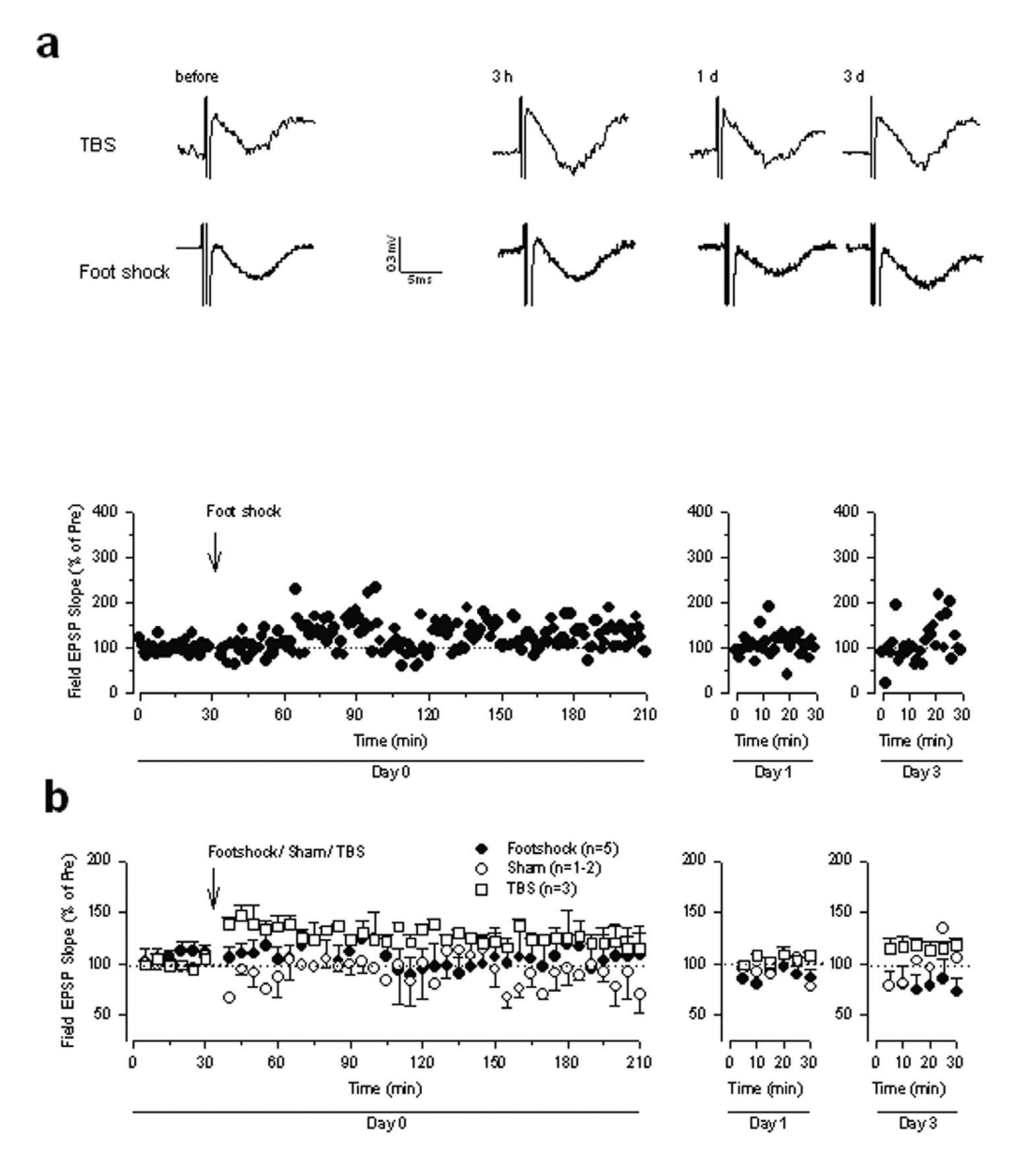
**Fear conditioning did not cause long-term plasticity in ACC-ACC synapses. (a) **Evoked fast responses in the ACC by stimulation applied to the other side of ACC were not affected by single foot shock that induced classic fear memory. However, TBS induced synaptic potentiation in the ACC. Insets: Traces of evoked responses before, 3 hour, 1 day and 3 days after fear conditioning.**(b) **Summarized data of experiments shown in (a) and mice receiving the treatment without the foot shock (n = 2 mice).

What about the possibility that synaptic transmission within the ACC may contribute to memory formation as those reported in the amygdala? We suspect that synaptic changes between two sides of the ACC connected by callosal projection fibers may serve as an ideal candidate for the storage of such information, since the thalamic-cortical connections are important for ascending nociceptive sensory transmission. In order to detect possible changes in the ACC, we performed recordings of synaptic responses in the ACC before and after fear conditioning. As shown in Fig. 9, we found that synaptic responses to stimulation of the callosal projection fibers from the other side of the ACC were not affected by fear conditioning (n = 5 mice). Furthermore, measurements of the same responses at one day and 3 days after fear conditioning also did not reveal any significant changes (Fig. 9), indicating that synaptic transmission between the ACCs did not undergo synaptic potentiation after fear conditioning. To test if synaptic transmission may undergo potentiation by theta burst stimulation, we also tested responses in freely moving mice after applying theta burst stimulation (TBS) locally into the ACC (n = 3 mice). As shown in Fig. 9, we found that synaptic transmission was enhanced for the initial 3 hours.

## Discussion

We present strong evidence that the ACC serves as a critical region for pain unpleasantness in the brains of adult mice. Our results are consistent with previous reports from human imaging that show that ACC is important for pain affect or unpleasantness [2,3,5]. Clinical reports show that lesions in the human ACC selectively influence the unpleasant component of pain [9,10]. In animals, lesions in the ACC blocked formalin-induced conditioned place avoidance [26]. Both animal and human studies indicate that neurons in the ACC respond to peripheral painful stimuli or electric shocks [6,28], and injury triggered activation of immediate early genes in the ACC [27,29]. Unlike neurons in the somatosensory cortex, neurons in the ACC tend to have widely diffuse receptive fields, and often contain the whole body of animals, supporting its role in coding unpleasantness of pain. Our results using fear memory to measure pain triggered by ACC stimulation avoid the possible contribution of descending modulation to commonly used behavioural nociceptive responses [33-37]. The present study provides a new approach to study the role of pain in higher brain function.

The ACC is a complex and heterogeneous cortex. Neurophysiological recordings, neuropsychological tests and human imaging studies suggest that the ACC plays a key role in cognitive control and is involved in response conflict monitoring [12,14,19]. The ACC may be involved in the neural representation of motivational drives, including sexual desire, hunger and the motivational aspect of pain [13,22]. However, synaptic and molecular mechanisms of the ACC in these higher order functions are largely unknown, due to the lack of animal models. The different roles of the ACC in various functions require caution when explaining the current results. One possible explanation is that ACC neurons express pain affect, general unpleasantness, aspects of cognition and motor response, and pain analgesia. These different functions might be topographically organized along the extent of the ACC, and through its potential interactions with other cortical areas. Furthermore, possible differences between mouse and human brains certainly contribute to some of the different reports on the functions of human ACC vs mice as presented here. For example, Shidara and Richmond [15] reported that neural activity in the ACC codes the degree of reward expectancy [15]. It is unclear if any of those neurons are also involved in encoding pain unpleasantness. Due to the important role of the ACC in attention and anticipation, another possibility is that ACC stimulation alters learning by affecting such processes. Specifically, the ACC stimulation produces some anticipatory "state" for unpleasantness rather than one of aversion or unpleasantness. To test this possibility, we performed experiments using a newly developed pain-related hot-plate escaping test. Chemical activation of excitatory mGluRs in the ACC enhanced the escape responses. These results suggest that enhanced escape responses and behavioural freezing responses are most likely due to pain-related unpleasantness, and are unlikely to be explained by simply the cessation of activity or goal directed activity. It is also possible that ACC stimulation only causes pain, and the unpleasantness is coded in other regions of the brain. For fear memory, the projection from the ACC to the amygdala plays an important role. Future experiments are clearly needed to address these possibilities. Interestingly, a recent study from Johansen and Fields [53] showed that excitatory amino acid microinjection into the ACC in rats during conditioning produced avoidance learning in the absence of a peripheral noxious stimulus. These findings indicate that that neurons in the ACC of adult rats and mice mediate both pain-induced negative affect and a nociceptive aversive teaching signal (see [53]).

Insular cortex has recently been shown to contribute to pain perception [38,54]. It was shown that the insular cortex may tonically control spinal nociceptive transmission through descending inhibitory systems [54]. By contrast, it has been reported that stimulation in the ACC produced no antinociceptive effect, and facilitated spinal nociceptive tail-flick reflex [33]. Inhibition of excitatory transmission in the ACC by microinjection of opioids produced powerful analgesic effects in freely moving animals [25], suggesting that the ACC is unique as a centre for unpleasantness. Recent studies in knockout mice for adenylyl cyclases1 and 8 as well as transgenic mice over expressing NR2B receptors consistently indicate that activity-dependent plastic signaling pathways in the ACC may contribute to persistent pain, a classic condition with long-term pain-related unpleasantness [29,30]. One possibility is that ACC stimulation may activate endogenous analgesia from the ACC, in the absence of nociception, might itself be aversive. To test this possibility, we performed experiments in awake mice and rats using the spinal nociceptive tail-flick reflex, a classic behavioural test for endogenous analgesic/antinociceptive systems [33]. Activation of mGluRs in the ACC by tACPD actually facilitated behavioural responses in both the tail-flick reflex and hot-plate tests, providing direct evidence that endogenous facilitatory but not inhibitory systems are activated. Moreover, intrathecal injection of a serotonergic receptor antagonist methysergide that blocked descending facilitatory modulation from the RVM [34,38] completely blocked the facilitatory effects in awakened or anaesthetized rats. These findings thus provide the first evidence, to our knowledge, in freely moving animals, excitatory activity in the ACC exert descending facilitatory modulation on spinal nociception

Experiments using lesions in the central nervous system provide important evidence for the involvement of certain structures in learning and memory [55]. However, when using the lesion technique it is difficult to distinguish between roles of the ACC in the expression of freezing responses, or the formation of fear memory. In the present study, we performed a reversible blockade of ACC activity during fear conditioning and found that fear memory was blocked. Our results indicate that the blockade of fear memory is not simply due to the ACC-dependent expression of freezing. One possible mechanism for the roles of the ACC in fear memory is that inputs from the ACC feedback to the amygdala for the formation of fearful memory. Indeed, many ACC neurons directly project to the amygdala [56,57]. We found that auditory fear memory induced by pairing the ACC stimulation with a tone was blocked by bilateral injection of the NMDA receptor antagonist AP-5 into the amygdala. Contextual memory was not significantly affected, suggesting that other structures may be involved or required for the expression of contextual fear memory [55]. Our results provide new evidence that cortical input from the ACC (coding unpleasantness) is critical for the formation of fear memory (see Fig. 7e). Due to limitations of the microinjection technique, we cannot completely rule out the possible diffusion of AP-5 into other nuclei within the amygdala. Finally, we believe that mouse genetic models will provide ample opportunities for us to explore the molecular mechanisms for high-order brain functions in experimental conditions. Together with imaging and electrophysiological studies in humans and primates, we hope to reveal new central and molecular targets for the treatment of central pain, phantom pain and the unpleasantness related to various mental disorders.

## Methods

### Animals

Animals were adult male C57BL6/J mice or Sprague-Dawley albino rats that were housed individually and maintained on a 12/12 h light/dark cycle. Food and water were provided *ad libitum*.

### Brain electrode and microinjection cannula implantation

Animals were anesthetized with sodium pentobarbital (80 mg/kg, i.p.) and implanted unilaterally with a tungsten electrode in the ACC (0.62 mm anterior, 0.4 mm lateral and 1.7 mm ventral to the Bregma) or S1 (0.62 mm anterior, 2.8 mm lateral and 2.3 mm ventral to the Bregma) [58] under aseptic conditions. The tungsten wire extended 0.1–0.2 mm of the guide cannula that was used as a reference. Mice were allowed to recover for 2 weeks before the experiments. In preliminary experiments, we performed several experiments to locate electrodes into the mouse ACC. All procedures were in accordance with the Animal Studies Committee at the University of Toronto. At the end of the experiment, using standard histological methods, 30 μm brain sections were stained with cresyl violet and examined by light microscopy for electrode placements or cannula penetrations. We only used animals with stable implantation of electrodes that did not exhibit abnormal behaviour during surgical recovery. Results from stimulation sites outside of the ACC were not used in the current studies.

### Behavioral conditioning

On the day of conditioning, the electrode assembly was connected to the stimulating hardware under brief isoflurane sedation. Mice were allowed 5 min to recover and habituate in the mouse conditioning chamber located in a sound-attenuating box (Med Associates). A commutator (CRIST INSTRUMENT CO.) was used to handle the connecting wires while mice were moving. Each mouse received 3 pairings of training with 1 min in between. The conditioned stimulus (CS) was a tone (2.8 kHz, 85 dB sound pressure level, 30 s), and the unconditioned stimulus (US) was the 10 s ACC stimulation that co-terminated with the tone. The electrical stimulation parameters are as following: 0.3 mA, 0.2 ms pulse duration, 5 pulses at 100 Hz per train, 200 ms train interval. 1 hour, 1 day and 3 days later, animals were first exposed to the conditioning context without tone for 3 min and then to a novel context without tone for 3 min followed by 3 min with tone presentation. Freezing responses was scored visually and presented as percentage of the total period of time observed [31]. Baseline responses were subtracted in order to evaluate responses to the context or tone. For the unpaired training, tone and ACC stimulation were delivered randomly with 40 s – 80 s interval. In some experiments, tACPD (0.25 μg in 0.5 μl) was injected into the ACC unilaterally. After the injection, mice were placed in the conditioning chamber. Each mouse received a tone (see above) for 10 min (at 0–5 and 10–15 min after the tACPD injection).

### Pharmacological treatment

Under anesthesia of sodium pentobarbital (80 mg/kg), 25-gauge guide cannulas were implanted bilaterally into the ACC (0.62 mm anterior to Bregma, 0.5 mm lateral from the midline, 0.9 mm beneath to the surface of the skull) or BLAC (1.94 mm anterior to Bregma, 3.5 mm lateral from the midline, 4.2 mm beneath to the surface of the skull). Mice were given at least 2 weeks for recovery after cannula implantation. 30-gauge injection cannula was 0.8 mm lower than the guide. For intra-ACC infusion, 0.5 μl muscimol (1 μg/μl) or saline was delivered bilaterally within 90 s using a pump. 15 min later mice were conditioned by 1 pairing of a tone (2.8 kHz, 85 dB, 30 s) and a foot shock (0.75 mA, 2 s) that terminated simultaneously with the tone. For intra-BLAC infusion, 0.5 μl AP5 (2 μg/μl) or saline was delivered bilaterally within 90 s. 15 min later mice received 3 pairings of tone and ACC stimulation, exactly as above.

### Hot-plate escape test-a new behavioral test

Adult mice were trained using a modified thermal hot-plate (10" × 10" heating surface) (Columbus Instruments, Columbus) maintained at 50°C, with an escapable non-thermal platform (8" × 5.5" surface). During the first training trial, the escape platform was blocked until the mice showed signs of nociception (e.g., licking of the hind paws). The escape route was then unblocked, and the mice were then free to explore both platforms. The first time entry to the escape platform was recorded. The total duration of each training trial was 3 min. Mice were returned to the same modified thermal hotplate one day after training, with the escape route remaining open, and the temperature set at a room temperature of 22°C. The first time entry to the escape platform was recorded, and the mice were returned back to their home cage upon escape. The maximum test time was 3 min. Mice were tested a total of eight times with an inter-trial interval of 30 min. Mice that remained on the hotplate for the total test duration were recorded with having an escape time of 3 min.

### Spinal nociceptive tail-flick reflex

Mice or rats were used either in the awake or halothane-anesthetized states. The spinal nociceptive tail-flick reflex was evoked by noxious radiant heat provided by a 50 W projector lamp focused on a 1.5 × 10 mm area on the underside of the tail. The latency to reflexive removal of the tail from the heat was measured by a digital photocell timer to the nearest 0.1 s. Baseline TF latency was the mean of 3 trials taken at 3 min intervals. For experiments using anesthesia, rats were anesthetized with 2–3% halothane (Ohio Medical Products) delivered via a specialized gas adapter (Stoleting Instrument; IL) with 30% O_2 _balanced with nitrogen. Body temperature was maintained at 37 ± 0.5°C by a circulating water, thermostatically-controlled heating pad.

### Chemical microinjection into the ACC

A mGluR agonist, trans-(±)-1-amino-(1S, 3R)-cyclopentanedicarboxylic acid (tACPD), was microinjected into the ACC in a volume of 0.5 μl via an injection cannula (33-gauge, 0.20 mm O.D.) inserted through the 26-gauge guide cannula and also extending 2 mm beyond its tip. Injection of tACPD was monitored by following the movement of an air bubble in a length of calibrated tubing between the syringe and the cannula.

### Intrathecal drug injection

Intrathecal catheters (PE-10 tubing, 8.5 cm in length) were inserted through a small opening in the cisterna magna and extended to the lumbar subarachnoid space. In case of experiments in freely moving rats, animals were recovered for at least two weeks before the testing. Intrathecal drug administration was done in the awake or halothane-anesthetized rats. In rats with ACC drug microinjection, baseline TF latencies were determined at 3, 6 and 9 min before and after tACPD injection. After observing the facilitatory effects at 10 min after the injection, methysergide or saline were injected intrathecally to examine if the facilitatory effects may be blocked. The selection of methysergide and dose used are based on previous studies of descending facilitatory modulation from the RVM to the spinal cord [33,38].

### Electrophysiological recordings in freely moving mice

Mice were anesthetized with sodium pentobarbital (80 mg/kg, i.p.). A concentric stimulating electrode was positioned in the right ACC and another concentric recording electrode was placed to the left ACC (both were in Cg2). Dental cement was used to keep the electrodes in place for the recordings of field potential in freely moving mice. Following surgery, mice were allowed to recovery for at least two weeks. Test responses were elicited by monophasic stimuli (200 μs, 75–190 μA, 1/60 s) at an intensity that evoked 40–50% of the maximal responses. fEPSP potentiation is expressed as percentage change relative to the mean baseline response during the 30 min prior to the single footshock stimulation.

### Data analysis

Data are presented as mean ± 1 standard error of mean (S.E.M). Facilitation of the TF reflex or hot-plate test is presented as a percentage of the control TF latency. Results were expressed as mean ± s.e.m. One-way ANOVA or two-way ANOVA with repeated measurements was used to compare the differences between treatments. If not stated otherwise, post-hoc comparisons were made with Tukey test. In all cases, p < 0.05 was considered statistically significant.
